# Using Positively Charged Magnetic Nanoparticles to Capture Bacteria at Ultralow Concentration

**DOI:** 10.1186/s11671-019-3005-z

**Published:** 2019-06-04

**Authors:** Zhiming Li, Jinyuan Ma, Jun Ruan, Xuan Zhuang

**Affiliations:** 1 Institue of Reproductive Health, Tongji Medical College, Huazhong University of Science and Technology, Wuhan, 430030 Hubei China; 2 Department of Urology, the First Affiliated Hospital of Xiamen University, Xiamen, 361003 Fujian China; 3 Department of Pharmacy, Shanghai Dermatology Hospital, Shanghai, 200443 China; 4 College of Life Sciences, Central China Normal University, Wuhan, 430079 China; 5 Department of Clinical Medicine, Fujian Medical University, Fuzhou, 350005 Fujian Province China

**Keywords:** Bacterial charge, Electrostatic attraction, Magnetic nanoparticles, *E. coli*

## Abstract

Detecting bacteria at low concentrations without time-consuming culture processes would allow rapid diagnoses. Since electrostatic attraction exists between negatively charged bacterial cells and positively charged magnetic nanoparticles (NP+), capture of bacteria holds great promise towards achieving this goal. Here, we present a rapid and highly efficient approach to capture *Escherichia coli*, which was used as a model for gram-negative bacteria. Capture of *E. coli* at very low concentrations of 10 and 100 CFU/mL using NP+ is rapidly and efficiently achieved within 1 h. Moreover, the capture efficiency of NP+ was over 90% by analyzing the number of bacterial colonies on the plate. Optical and transmission electron microscopy confirmed the bacterial capture abilities of electrically charged nanoparticles (NPs). In contrast, negatively charged magnetic nanoparticles (NP−) did not show affinities towards *E. coli*. These results showed that bacterial cells, such as *E. coli*, carry a negative charge. Unlike a ligand-dependent capture system, our designed NP+ has potentials to capture a broad range of bacteria via electrostatic attractions.

**Electronic supplementary material:**

The online version of this article (10.1186/s11671-019-3005-z) contains supplementary material, which is available to authorized users.

## Background

Infectious diseases are among the world’s most pressing health challenges. Microbial contamination of water resources is a major threat to public health. *Escherichia coli* (*E. coli*), a gram-negative bacterium, is very common in contaminated water and food. Some strains of *E. coli* can even cause serious bacterial infections. Bacteria at low concentrations are difficult to detect and usually require a pre-enriching process before further analysis. Culture-based microbiological methods are laborious and may take several days. Additionally, some bacterial strains may enter a viable but non-culturable state where they are viable but not culturable on routine agar, which impedes their detection by culture-based methods [1]. Inversely, rapid capture and decontamination of bacterial pathogens could provide real-time results to mitigate infectious disease outbreaks.

A variety of materials are developed for rapid capture and removal of bacteria from the contaminated source. Carbon nanotubes and resin-linked oligoacyllysine bead have been used to remove the bacteria from water [2, 3]. Magnetic nanoparticles, which can be conveniently separated from various resources by the employment of magnetic process, were widely used for bacteria detection and decontamination after functionalized with organic molecules [4–6]. The magnetic-based techniques have the advantages of target capture by time-saving (common separation time within 1 h), high recovery, possible automation, and scale-up separation [7]. The efficiency and selectivity of magnetic separation largely depends on the ligands, but sometimes it is hard to obtain a ligand with high affinity and specificity to the target. Therefore, it is necessary to develop a bacterial capture system with ligand-independent magnetic nanoparticles to capture the bacteria, especially under low concentrations.

Many scientists have investigated the nature of the electric charge of bacteria. Bechhold (1904) was the first to find the fact that bacterial cells carry a negative charge [8]. While it was already known that the large populations of bacterial cells tended to maintain a negative charge, little is known about the electrophysiology of bacteria at the level of single cells. In 2011, Cohen et al. revealed electrical spiking in *E. coli* at up to 1 Hz using a fluorescent voltage-indicating protein [9]. Since many kinds of bacterial cell walls are negatively charged, positive charged nanoparticles can strongly interact with a broad spectrum of bacteria via electrostatic interactions.

To take advantage of magnetic nanoparticles and negative charge of individual bacteria for fast pathogen detection, we designed a system to capture bacteria under low concentrations. Positively charged magnetic nanoparticles were fabricated by polyethylenimine (PEI), which is composed of abundant amine groups. Then we investigated the affinity of PEI functionalized nanoparticles against *E. coli*. This innovative method provides efficient binding to the bacteria by electrostatic interactions.

## Materials and Methods

### Nanomaterials

Iron (III) chloride hydrate (FeCl_3_·6H_2_O), ammonium hydroxide (NH_4_OH, 28 wt%), hydro-chloric acid (37 wt% aqueous solution), ethylene glycol, and sodium acetate were purchased from Shanghai (China) Reagent Company. Tetraethyl orthosilicate (TEOS), (3-aminopropyl) triethoxysilane (APTES), and fluorescein tetramethylrhodamine (TRITC) were purchased from Sigma-Aldrich (USA). Branched poly(ethylene imine) (PEI, 99%, *Mw* =10,000) was purchased from Alfa Aesar. All the solutions were prepared using Milli-Q deionized water (18.2 MΩ cm at 25 °C resistivity).

### NP Syntheses

Fe_3_O_4_ nanoparticles were prepared by a solvothermal reaction [10]. Briefly, 0.081 g of FeCl_3_·6H_2_O was dissolved in 30 mL of ethylene glycol under magnetic stirring. Then, 0.3 g of polyacrylic acid (PAA) and 1.8 g urea were added to this solution. After being stirred for 30 min, the solution was heated at 200 °C for 12 h by using a Teflon-lined stainless-steel autoclave. When cooled to room temperature, a black product, namely magnetic nanoparticle cores, was collected by a magnet. Followed by washing with ethanol and deionized water each three times, the Fe_3_O_4_ nanoparticles were treated with 0.15 M HCl under sonication for 15 min and then were coated with silica via hydrolysis and TEOS.

To prepare the negatively charged fluorescent magnetic nanoparticles (NP−), APTES-TRITC (C33H44N3O6Si) complex was first reacted under dark conditions overnight in ethanol. The complex was then grafted to the Fe_3_O_4_ nanoparticles through reaction between APTES and hydroxyl groups on the Fe_3_O_4_@SiO_2_ nanoparticle. Subsequently, 30 μL of TEOS was added and reacted for 24 h in the dark. Followed by washing with ethanol and deionized water each three times, fluorescent NP− were produced. Through the modification of NP− with the polycation polymer PEI, the positively charged magnetic nanoparticles (NP+) were finished.

### NP Characterization

Transmission electron microscopy (TEM) studies were performed by a TECNAI F^− 30^ high-resolution transmission electron microscope operating at 300 kV. The particle size and zeta potential of NPs were determined by Malvern Zeta Sizer Nano series (Westborough, MA). Fluorescence was examined with a Carl Zeiss LSM5 EXITER laser scanning confocal microscope (Zeiss, Jena, Germany).

### Bacteria Preparation

The gram-negative strain *E. coli* BL21 were used as the model bacteria. *E. coli* was cultivated in 100 mL of Luria Broth growth medium [11]. The bacteria were cultured in a thermostatic incubator at 200 rpm, 37 °C for 16 h. Subsequently, 100 μL of the bacterial culture was removed, diluted with the medium 1 × 10^5^ times, coated on the agar, and cultured at 37 °C for 24 h. The number of colony-forming units was counted. The remaining bacterial cells were harvested by centrifugation at 5000 rpm for 5 min, washed thrice with 1× PBS (10 mM, pH 7.4), and diluted to concentrations of approximately 1 × 10^3^ colony-forming unit (CFU)/mL. For safety considerations, all of the bacterial samples were placed in an autoclave at 121 °C for 20 min to kill bacteria before disposal and all glassware in contact with the bacteria was sterilized before and after use.

### Bacteria Capture Experiment

All the batch capture studies were conducted in sterilized 1× PBS buffer. Forty microliters of NPs (1 μg/μL) were dispersed in the sterilized saline under ultrasonication for 10 min, and then 1 mL of the bacterial solution (approximately 10^3^ CFU/mL) was added into the suspension. After incubation of 10 min, the NP-bounded bacteria were captured via a permanent magnet onto the wall of the vial, and free bacteria were removed with the wash solution. The captured bacteria were released by removing the magnet and resuspended in PBS. For microscopic analysis, an aliquot of bacteria was spread onto slides and stained with Hema-3 (Fisher Diagnostics). For immunofluorescence analysis, an aliquot of bacteria was spread onto slides and stained with 4′-6-diamidino-2-phenylindole (DAPI).

### Bacterial Capture Efficiency of NPs at Different Concentrations

One milliliter of bacterial suspension (approximately 2 × 10^2^ CFU/mL) was incubated with different amounts (5, 10, 20, 30, 40, 50, 75, and 100 μg/mL) of NP+ or NP− for 10 min. After magnetic separation by the nanoparticles, total solution was then sampled and analyzed for bacterial concentration via a plate counting method. The bacterial-capture efficiency of the NPs was tested by counting the number of CFU on the LB-agar plates.

### Capability of NPs to Capture Bacteria at Low Concentrations

Forty micrograms of NP+ or NP− was incubated with 1 mL of bacterial suspension at very low concentrations (10 and 10^2^ CFU/mL). After magnetic separation by the nanoparticles, total solution was then sampled and analyzed for bacterial concentration via a plate counting method. The bacterial-capture efficiency of the NPs was tested by counting the number of CFU on the LB-agar plates.

### Statistical Analysis

Results were expressed as mean ± standard deviation (SD) as indicated in the figure legends. A two-way analysis of variance (ANOVA) with proper hoc analysis was calculated using GraphPad Prism software with *P* values < 0.05 considered statistically significant.

## Results

### Characterization of Magnetic NPs

**Fig. 1:**
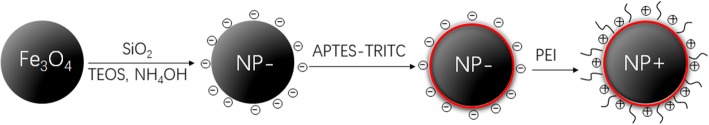
Design of the nanoparticles. Schematic diagram showing the design of surface-charged, fluorescent, superparamagnetic composite nanoparticles (NPs)

The schematic diagram for preparation of the surface-charged magnetic composite nanoparticles is displayed in Fig. 1. The Fe_3_O_4_ nanoparticles are conjugated with APTES to form a thin layer of SiO_2_ shell on the surface of nanoparticles upon reaction with TEOS and NH_4_OH. To visualize and quantify captured cells directly, the APTES-TRITC complex is initially reacted, followed by grafting onto the surface of the Fe_3_O_4_@silica composites through a classical sol-gel reaction. Abundant SiOH groups govern the overall surface of this product, exhibiting a strong negative surface charge, namely negatively charged magnetic nanoparticles (NP−). For positively charged magnetic nanoparticles (NP+), PEI molecules are used to cover and modify the surface of NP−. The modified product shows a strong positive surface charge due to the abundant presence of amine groups.

**Fig. 2:**
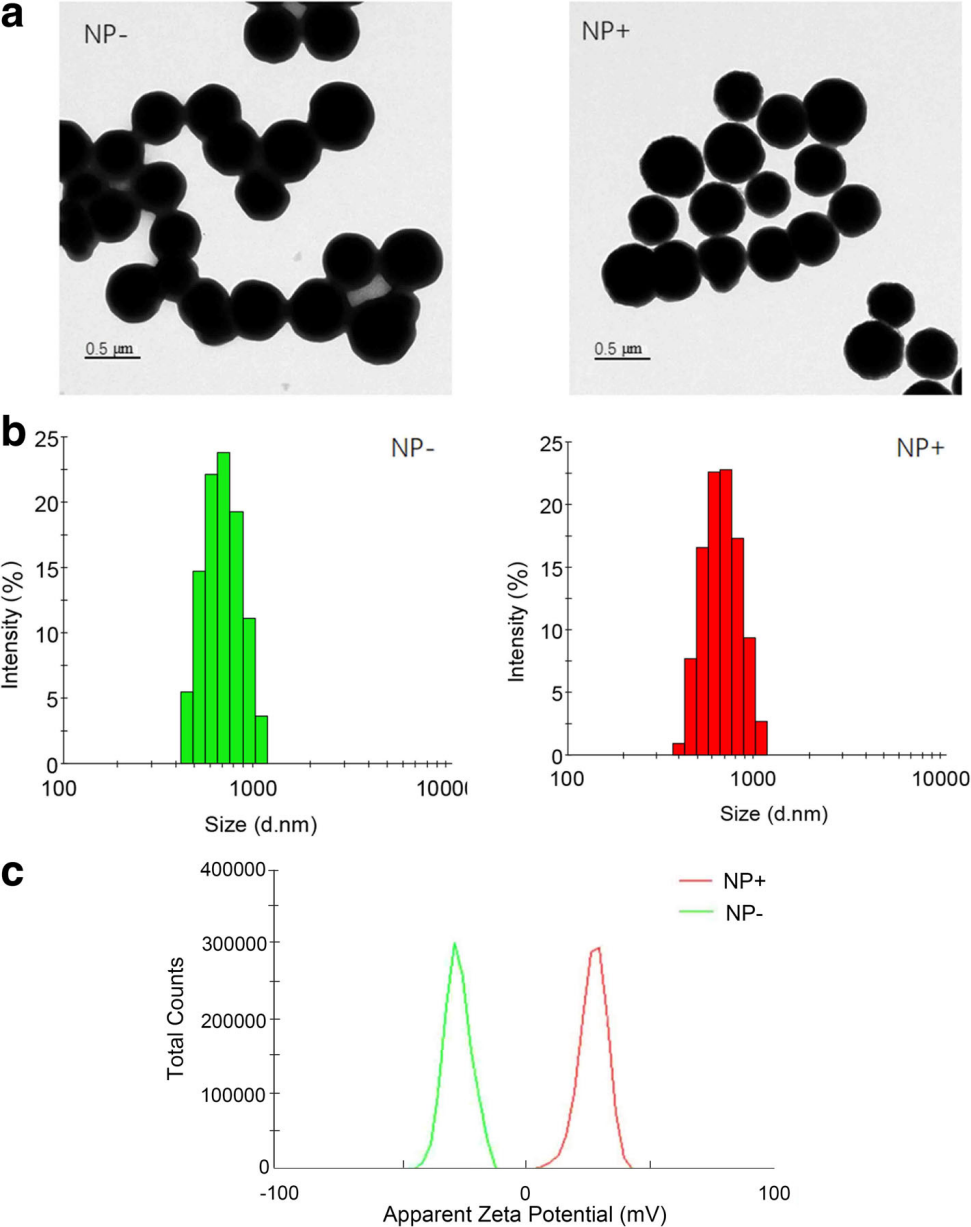
Characterization of the nanoparticles. **a** Transmission electronic microscopy (TEM) image of positively charged nanoparticles (NP+) and negatively charged nanoparticles (NP−). **b** Dynamic light scattering size and distribution of NPs. **c** Zeta potential distributions of NPs

As shown in Fig. 2a, TEM demonstrated that magnetic composite nanoparticles had a diameter of 450 nm, which were composed of uniformed SiO_2_ coating (size of 60 nm). Dynamic light scattering (DLS) of the particles in Fig. 2b displayed a narrow size distribution with an increased average diameter after surface functionalization. The maximum sizes of the composite nanoparticles with the positive and negative charges are 620 and 700 nm, respectively. Nanoparticles measured by DLS are usually larger than those measured by TEM. This is because the DLS-assessed size is influenced by Brownian motion and depends on the ambient temperature, the dynamic radius of the nanoparticle, and the extent of nanoparticle agglomeration triggered by a static environment via the occurrence of confliction [12]. Figure 2c shows the zeta potential distributions of the negative and positive nanoparticles. In deionized water (pH 7.0), the zeta potentials of the NP− and NP+ are − 26.6 mV and + 28.1 mV, respectively. The pH dependences of zeta potentials for NP+ are depicted in Additional file 1: Figure S1. These results indicated that the surface-charged nanoparticles are well dispersed in aqueous solution under neutral conditions, which could be applied for cell capture.

### Ability of Magnetic NPs to Capture *E. coli*

**Fig. 3:**
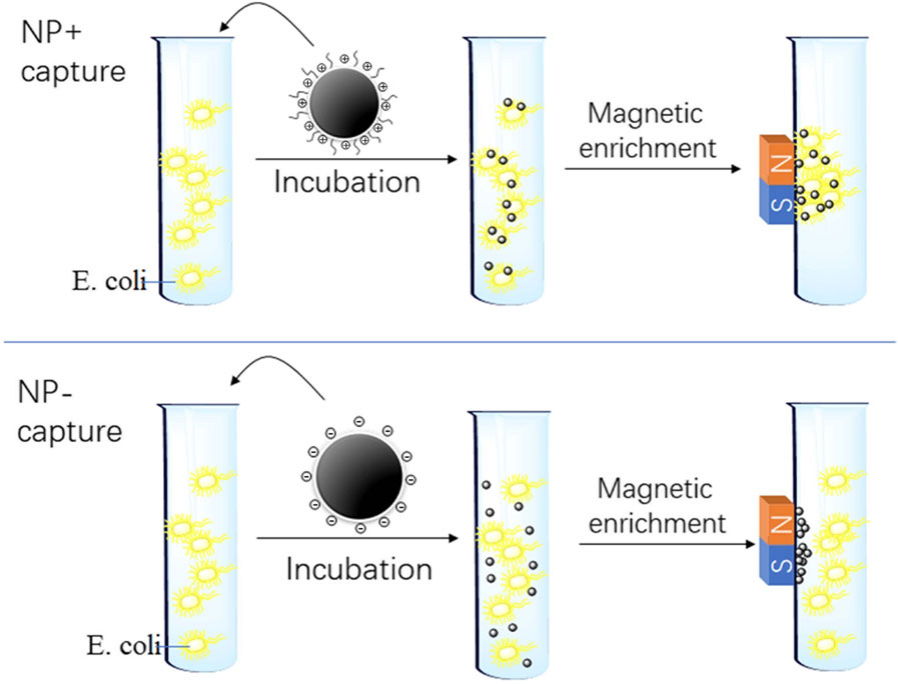
Illustration of the procedures of bacteria capture. *E. coli* in suspension are respectively mixed with NP+ and NP−, followed by 10 min incubation. The “magnetized” bacteria were attracted onto the wall of the vial by a magnet. After the removal of the remaining solution, bacteria were captured by NPs, washed using PBS, and counted from the number of the bacterial colonies

Figure 3 shows the general experimental procedure of bacteria capture. NPs were mixed with a solution of bacteria and incubated at room temperature for 10 min. Subsequently, we used a permanent magnet to capture the “magnetized” bacteria (magnetic nanoparticles bounded to the cell surface) onto the wall of the tube. After the removal of the remaining solution and washing the aggregates by PBS (with a magnet outside), we transferred the aggregates to a slide for microscopic analysis. As shown in the scheme, NP+ provides sufficient electrostatic responsiveness to quickly enrich *E. coli*. On the contrary, the bacteria are removed with rinsing PBS in the NP− experiment.

**Fig. 4:**
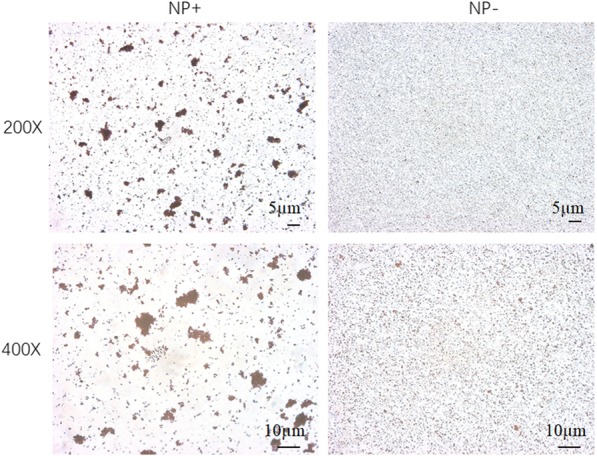
Comparison of *E. coli* binding capacity of NP+ and NP−. The left images are the phase-contrast fields of bacteria binding with NP+. The right images only have nanoparticles, suggesting that NP− without binding capacity to *E. coli* cells

**Fig. 5:**
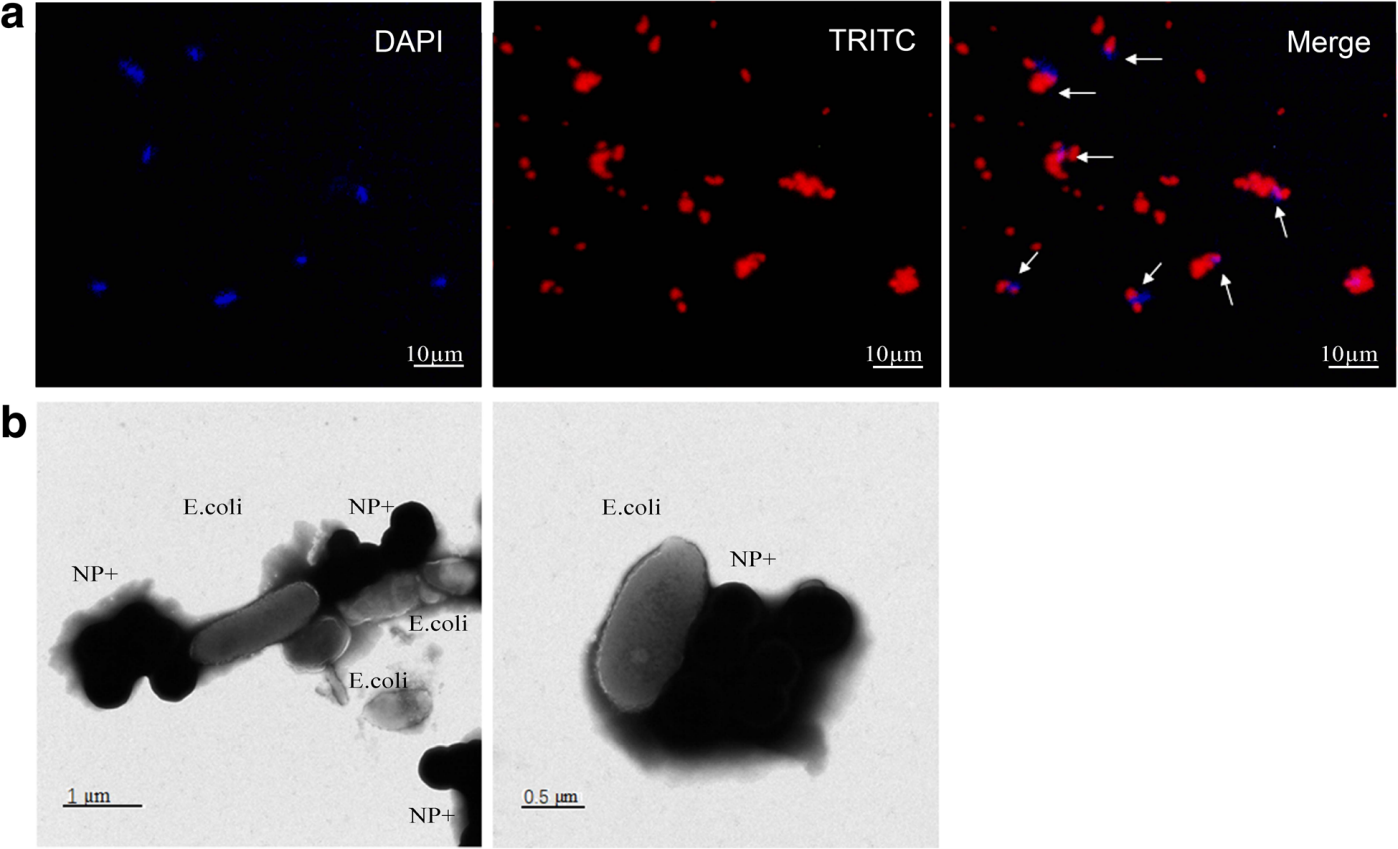
Fluorescent and TEM images of *E. coli* binding with NP+. **a** Upper panels show the fluorescent images of *E. coli* cells binding with NP+. DAPI is used to stain the cell nucleus. TRITC is labeled in NPs. **b** Lower panels are representative TEM images showing NP+ and *E. coli* complexes

Optical images for the nanoparticles are shown in Fig. 4. Optical image of NP− showed the monodisperse and uniformly distributed particles. However, NP+ tended to agglomerate. The size distribution of NP+ is obviously wider than that of NP−. These results demonstrated that NP+ and NP− had a completely different pattern of interaction with *E. coli*. To further investigate the bacterial affinity of NP+, the localization NP+ in *E. coli* was examined using fluorescence analysis. Figure 5a shows that captured *E. coli* are positive for both DAPI (blue color) and TRITC (red color). In order to clearly confirm the affinity of NP+ for bacteria, TEM technology was used. In Fig. 5b, a number of NP+ were observed to aggregate on the bacterial cell wall. These results suggested that NP+ have a strong affinity for bacteria.

### Detection of Low Concentrations of *E. coli*

**Fig. 6:**
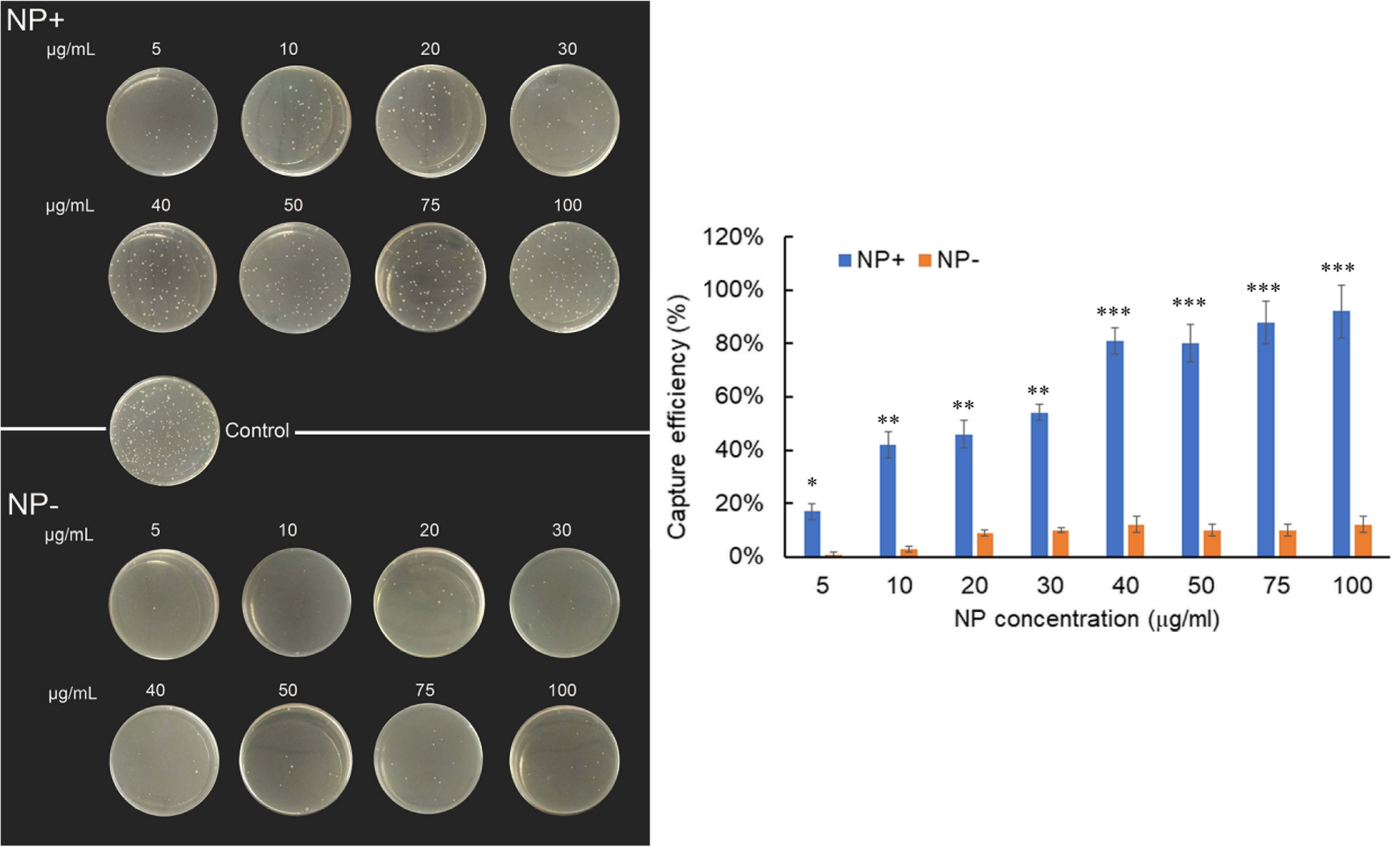
Capture efficiencies of *E. coli* by NP+ or NP− at various concentrations indicated. The left image is the photograph of LB-agar plates coated with *E. coli* captured by NP+ and NP−. The right image shows the bacterial capture efficiency of NP at different concentrations indicated. *E. coli* (2 × 10^2^ CFU in 1 mL PBS solution) without NP incubation was counted as 100% and served as control. *P < 0.05, **P < 0.01, ***P < 0.001

To further characterize the dynamics of the bacteria and NP+ interactions, we incubated *E. coli* at a constant number (2 × 10^2^ CFU/mL) with various concentrations of NPs ranging from 5 to 100 μg/mL. The NP-bound bacteria were then magnetically captured and separated. The magnetic capture efficiencies of bacteria by NP+ and NP− are plotted as shown in Fig. 6. The number of *E. coli* captured by NP− is only 12% even at a high concentration of 100 μg/mL. In contrast with NP−, NP+ showed significant bacterial capture capacities and achieved 81% of capture efficiency with 40 μg/mL (*P* < 0.001). As can be seen from the LB-agar plates, a dose-dependent increase of bacterial colonies with NP+ was demonstrated.

**Fig. 7:**
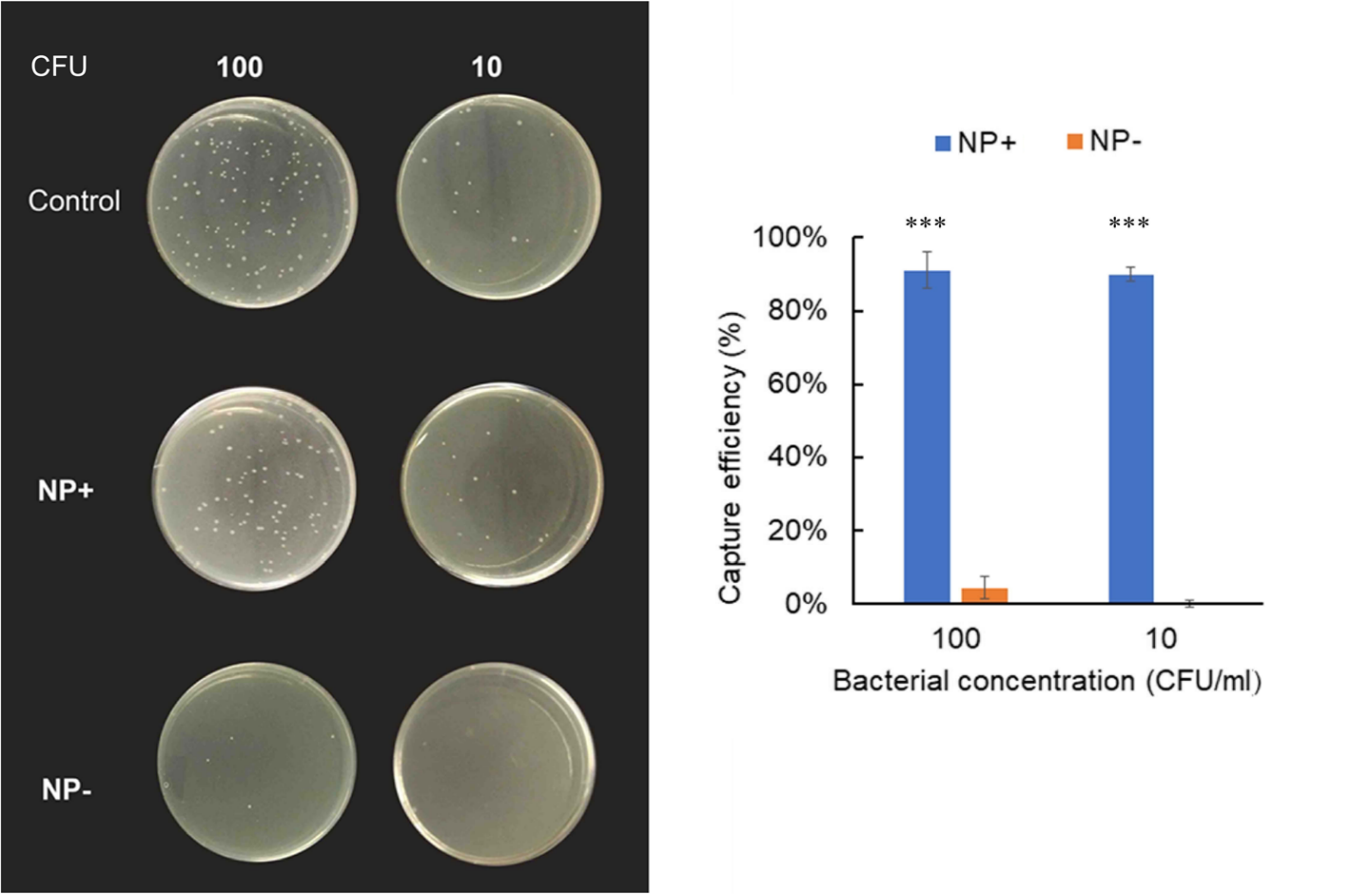
Capture efficiencies of *E. coli* by NP+ or NP− at ultralow concentrations. The left image is the photograph of LB-agar plates coated with *E. coli* captured by NP+ and NP−. The right image showed the *E. coli* capture efficiency of NPs (40 μg/mL) with ultralow concentrations of bacteria. *E. coli* (10 and 100 CFU in 1 mL PBS solution) without NP incubation was counted as 100% and served as control. ***P < 0.001

In order to confirm NP+ affinity for *E. coli* at ultralow concentration, we mixed 40 μg NP with 1 ml PBS solution containing only 10 and 100 CFU of *E. coli*. Figure 7 shows photographs of the resulting colonies in agar plates for all samples. As expected, the NP+ indeed captured *E. coli* at an ultralow concentration, while the bacterial colonies were not obvious in plates using NP−. The few colonies in the plate might be attributed to the non-specific affinity of nanoparticles. Further analysis indicated that over 90% bacterial capture efficiencies were obtained at an ultralow concentration (10 and 100 CFU/mL) using NP+. By contrast, the capture efficiency is less than 4% with NP− at the same conditions and has a significant difference (*P* < 0.001). These results suggest NP+ have a strong affinity for bacteria, which could be explained by the electrostatic attractions. To investigate the broad-spectrum bacterial capture properties, we employed three gram-positive bacteria (*Bacillus subtilis*, *Staphylococcus aureus*, and *Lactococcus lactis*) as models. As illustrated in Additional file 1: Figure S2, NP+ have a higher adsorption capacity for baclilli (*E. coli* and *B. subtilis*) than staphylococci (*S. aureus*) and streptococci (*L. lactis*). In addition, we also found that negatively charged molecules, such as 3-bromopyruvate (3-BP) and DNA, could interfere with the bacterial capture effect. The dead *E.coli* is invalid for such system (Additional file 1: Figure S3).

## Discussion

It is known that both gram-negative bacteria (such as, *E.coli*) and gram-positive bacteria (such as *B. subtilis*) much more easily interact with positively charged particles than negatively charged particles via electrostatic attractions [13, 14]. This was also found in our study. One advantage in our capture system is that the PEI-functionalized nanoparticles have more amine groups, which were able to capture bacteria at ultralow concentrations. To date, there is a few general and satisfactory assays that could detect bacteria at concentrations of less than 10^2^ CFU/mL without pre-enriching bacteria via a culture process. This study displayed a simple assay that uses electrically magnetic nanoparticles to capture and detect gram-negative bacteria (the organisms have a cytoplasmic membrane, a cell wall, and an intact outer membrane) within 1 h at a concentration of 10 CFU/mL.

In our experiment, we found that the surface charge of NPs can influence bacterial capture efficiencies. Here, the effects of charge at the surface of NPs on the bacterial capture efficiencies were studied using *E.coli* as a model bacteria. When NP+ were used in the capture assay, they exhibited efficient adsorptive ability of the bacteria. The bacterial capture efficiencies increased with the dosage of NP+. TEM microscopy shows macroscopic aggregates composed of nanoparticles and bacterial cells. In contrast, NP−, even at high concentrations, displayed low bacterial capture abilities. Overall, these observations demonstrated that the NP+ have a significantly higher capture ability than NP−.

Although gram-positive and gram-negative bacteria have differences in their membrane structure, most of them have a negative charge when cultivated at physiological pH values [15, 16]. Cell surface charge of bacterial cells has been characterized by electrostatic interaction chromatography (ESIC) [17]. The cell wall in gram-positive bacteria is mainly composed of a thick layer of peptidoglycan, which is embedded teichoic acid. On the other hand, the gram-negative bacteria have a layer of lipopolysaccharide at the external surface followed by a thin layer of peptidoglycan. The teichoic acid and lipopolysaccharides impart a negative charge to the surface of bacterial cells [18]. Previously, the positively charged silver nanoparticles (AgNPs) displayed the remarkable effectiveness against the microorganisms, including *E. coli* [19]. They found that the smaller particles are found to have greater antibacterial activity. We considered that larger particles may have a different benefit of bacterial absorbability. Firstly, larger particles difficultly reach the nuclear content of cells to cause the toxicity to the bacteria. Secondly, they can provide a greater surface area and therefore stronger bacterial interaction. Therefore, we designed and applied larger positively charged nanoparticles as a “sponge” agent to capture bacteria.

## Conclusions

In conclusion, by PEI-magnetic nanoparticles, we have demonstrated a simple and fast assay to allow *E. coli* to be captured and analyzed. The existing archives of optical and TEM profiles of bacteria allow easy identification of captured bacteria. The high recovery provided by positively charged magnetic nanoparticles will allow detection of other bacteria strains at ultra-low concentrations.

## Additional file

Additional file 1: **Figure S1.** The pH-dependent zeta potential and capture efficiency of the positive NPs. **Figure S2.** Effects of NP+ concentration on the capture efficiencies of four types of bacteria in PBS. **Figure S3.** Capture efficiency of the positive NPs at the different concentrations of 3-bromopyruvate (3-BP) (A), DNA (B), and the dead bacteria (C). (DOCX 424 kb)

## Abbreviations

APTES: 3-Aminopropyl triethoxysilane
CFU: Colony-forming unit
DLS: Dynamic light scattering
*E. coli*: *Escherichia coli*
NP: Negatively charged magnetic nanoparticles
NP+: Positively charged magnetic nanoparticles
NPs: Nanoparticles
PAA: Polyacrylic acid
PEI: Polyethylenimine
SD: Standard deviation
TEM: Transmission electron microscopy
TEOS: Tetraethyl orthosilicate
TRITC: Tetramethylrhodamine

## References

[1] Li L, Mendis N, Trigui H, Oliver JD, Faucher SP (2014). The importance of the viable but non-culturable state in human bacterial pathogens. Front Microbiol.

[2] Akasaka T, Watari F (2009). Capture of bacteria by flexible carbon nanotubes. Acta Biomater.

[3] Rotem S, Raz N, Kashi Y, Mor A (2010). Bacterial capture by peptide-mimetic oligoacyllysine surfaces. Appl Environ Microbiol.

[4] Huang YF, Wang YF, Yan XP (2010). Amine-functionalized magnetic nanoparticles for rapid capture and removal of bacterial pathogens. Environ Sci Technol.

[5] Wen CY, Hu J, Zhang ZL, Tian ZQ, Ou GP, Liao YL, Li Y, Xie M, Sun ZY, Pang DW (2013). One-step sensitive detection of Salmonella typhimurium by coupling magnetic capture and fluorescence identification with functional nanospheres. Anal Chem.

[6] Gu H, Ho PL, Tsang KW, Wang L, Xu B (2003). Using biofunctional magnetic nanoparticles to capture vancomycin-resistant enterococci and other gram-positive bacteria at ultralow concentration. J Am Chem Soc.

[7] Wang Y, Salazar JK (2016). Culture-independent rapid detection methods for bacterial pathogens and toxins in food matrices. Compr Rev Food Sci Food Saf.

[8] Olitzki L (1932). Electric charge of bacterial antigens. J Immunol.

[9] Kralj JM, Hochbaum DR, Douglass AD, Cohen AE (2011). Electrical spiking in Escherichia coli probed with a fluorescent voltage-indicating protein. Science.

[10] Li Z, Ruan J, Zhuang X (2019) Effective capture of circulating tumor cells using electrically charged magnetic nanoparticles. J Nanobiotechnol 17:59 10.1186/s12951-019-0491-1 PMC6499951 31054582

[11] Li Z, Chen S, Yang Y, Zhuang X, Tzeng CM (2018). Novel biomarker ZCCHC13 revealed by integrating DNA methylation and mRNA expression data in non-obstructive azoospermia. Cell Death Dis.

[12] Gebauer JS, Treuel L (2011). Influence of individual ionic components on the agglomeration kinetics of silver nanoparticles. J Colloid Interface Sci.

[13] Fang W, Han C, Zhang H, Wei W, Liu R, Shen Y (2016). Preparation of amino-functionalized magnetic nanoparticles for enhancement of bacterial capture efficiency. RSC Adv.

[14] Gottenbos B, Grijpma DW, van der Mei HC, Feijen J, Busscher HJ (2001). Antimicrobial effects of positively charged surfaces on adhering gram-positive and gram-negative bacteria. J Antimicrob Chemother.

[15] Wilson WW, Wade MM, Holman SC, Champlin FR (2001). Status of methods for assessing bacterial cell surface charge properties based on zeta potential measurements. J Microbiol Methods.

[16] Meadows PS (1971). The attachment of bacteria to solid surfaces. Arch Mikrobiol.

[17] Pedersen K (1981). Electrostatic interaction chromatography, a method for assaying the relative surface charges of bacteria. FEMS Microbiol Lett.

[18] Fedtke I, Götz F, Peschel A (2004). Bacterial evasion of innate host defenses–the Staphylococcus aureus lesson. Int J Med Microbiol.

[19] Abbaszadegan A, Ghahramani Y, Gholami A, Hemmateenejad B, Dorostkar S, Nabavizadeh M, Sharghi H (2015). The effect of charge at the surface of silver nanoparticles on antimicrobial activity against gram-positive and gram-negative bacteria: a preliminary study. J Nanomater.

