# Supplementary material for: Using Positively Charged Magnetic Nanoparticles to Capture Bacteria at Ultralow Concentration
Source: Nanoscale Res Lett. 2019 Jun 4;14:195. doi: 10.1186/s11671-019-3005-z (PMC6548795; doi:10.1186/s11671-019-3005-z)
Supplement: Supplementary file 1 — Figure S1. The pH-dependent zeta potential and capture efficiency of the positive NPs. Figure S2. Effects of NP+ concentration on the capture efficiencies of four types of bacteria in PBS. Figure S3. Capture efficiency of the positive NPs at the different concentrations of 3-bromopyruvate (3-BP) (A), DNA (B), and the dead bacteria (C). (DOCX 424 kb) [file 11671_2019_3005_MOESM1_ESM.docx]

Supplementary Material for

**Using positively charged magnetic nanoparticles to capture bacteria at ultralow concentration**

Zhiming Li, Jinyuan Ma, Jun Ruan, Xuan Zhuang


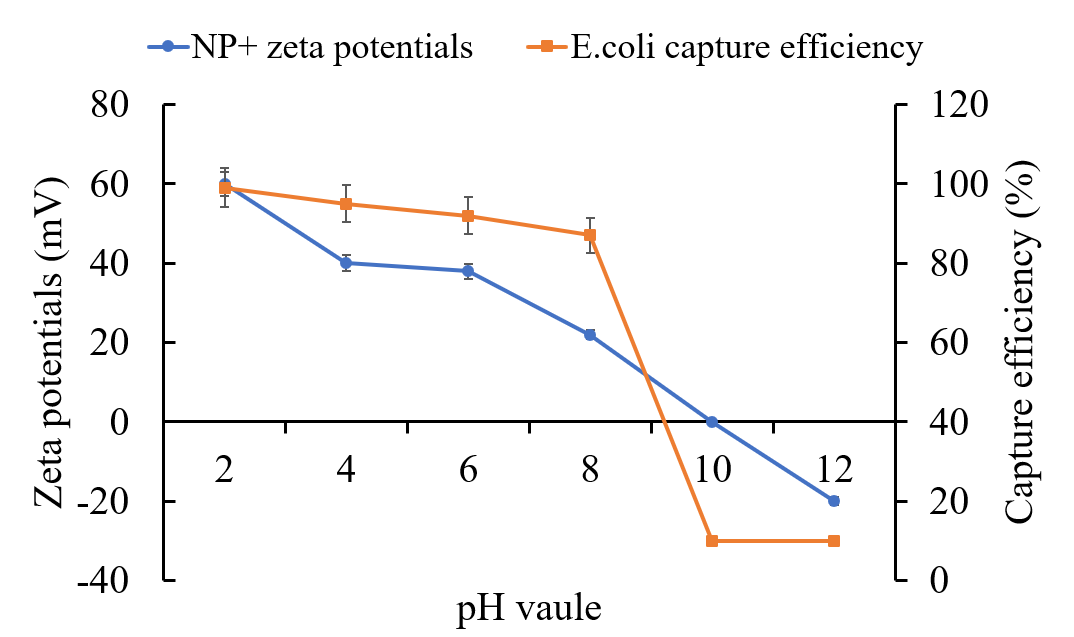


**Figure S1.** **The pH-dependent zeta potential and capture efficiency of the positive NPs.**

The zeta potentials of the positive charge of nanoparticles (NP+) were measured in PBS buﬀer (170 mM NaCl, 3.4 mM KCl, 15 mM phosphate, the pH values of solutions were adjusted by 1.0 M sodium hydroxide or hydrochloric acid) at diﬀerent pH values. Capture rate of *E. coli* was studied in PBS from pH 2.0 to 12.0. The optimal pH ranged from 2 to 8, when NP+ have the positive surface charges. The results were generally in agreement with the speculation that the capture of the bacteria by NP+ is driven by electrostatic force.


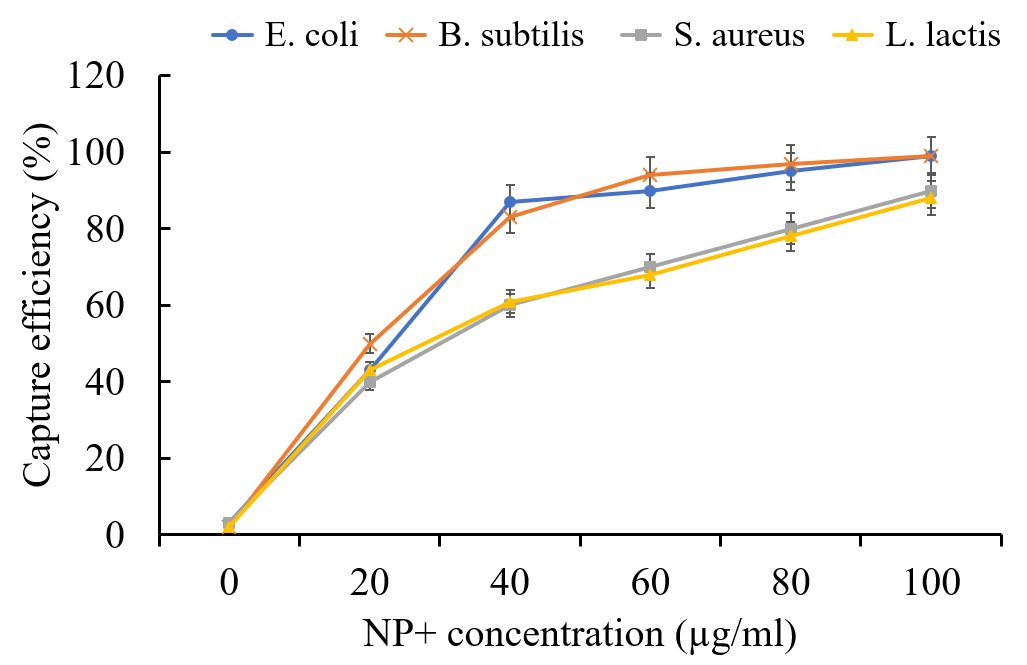


**Figure S2. Effects of NP+ concentration on the capture efficiencies of** **four types of bacteria in PBS.**

The capture efﬁciency of four bacteria was studied at a cell concentration of OD 600 =1.0. The capture rates increased with the dosage of NP+ in both gram-negative bacteria (*E.coli*) and gram-positive bacteria (*B. subtilis*, *S. aureus*, and *L.lactis*). The capture rate was 85.5% (*E.coli*), 81.3% (*B. subtilis*), 60.2% (*S. aureus*) and 61.7% (*L.lactis*), at a concentration of 40 µg/mL. It is interesting to note that NP+ have greater affinity to baclilli (*E. coli* and *B. subtilis*) than staphylococci (*S. aureus*) or streptococci (*L.lactis*).


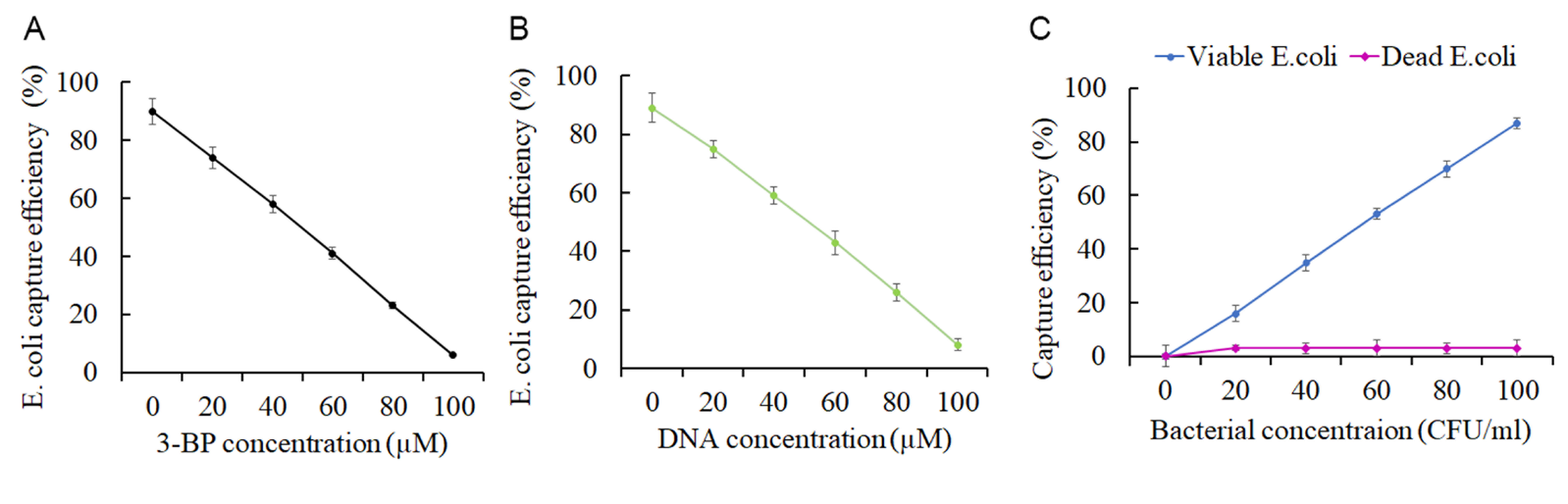


**Figure S3. Capture efficiency of the positive NPs at the different concentrations of 3-bromopyruvate (3-BP) (A), DNA (B), and the dead bacteria (C).**

We used 3-BP, DNA, and dead bacteria to vary the negative charges of the capture system. Under the same concentration of NP+ (40 µg/mL), capture eﬃciency of *E.coli* was investigated in the treatment of 3-BP, DNA, and the dead bacteria. 50% reduction is found when 50 µM 3-BP or 50 µM DNA is present. NP+ did not show the capture effect for the dead *E.coli* even at a high bacterial count of 100 CFU per mL. The viable *E.coli* were examined as a control experiment.
